# A risk of breast cancer in women - carriers of constitutional *CHEK2* gene mutations, originating from the North - Central Poland

**DOI:** 10.1186/1897-4287-12-10

**Published:** 2014-04-08

**Authors:** Aneta Bąk, Hanna Janiszewska, Anna Junkiert-Czarnecka, Marta Heise, Maria Pilarska-Deltow, Ryszard Laskowski, Magdalena Pasińska, Olga Haus

**Affiliations:** 1 Department of Clinical Genetics, Collegium Medicum, Nicolaus Copernicus University, Bydgoszcz, Poland; 2 Oncology Center, Prof. Franciszek Łukaszczyk Memorial Hospital, Bydgoszcz, Poland; 3 Department of Hematology, Blood Malignancies and Bone Marrow Transplantation, Medical University, Wrocław, Poland

**Keywords:** Breast cancer, Constitutional *CHEK2* mutation, Breast cancer familial aggregation

## Abstract

**Background:**

Germline mutations of the *CHEK2* gene have been reported to be associated with breast cancer. In this study, we analyzed the association of *CHEK2* mutations with the risk of development of breast cancer in women of North-Central Poland.

**Methods:**

420 women with breast cancer and 435 controls were tested for three protein truncating (IVS2 + 1G > A, 1100delC, del5395) and one missense (I157T) *CHEK2* mutation. IVS2 + 1G > A and I157T mutations were identified by RFLP-PCR, 1100delC variant was analyzed using an ASO-PCR and del5395 mutation by multiplex-PCR. The statistical tests: the odds ratio (OR) and Fisher’s exact test were used.

**Results:**

In 33 out of 420 (7.9%) women consecutively diagnosed with breast cancer, we detected one of four analyzed *CHEK2* mutations: I157T, 1100delC, IVS2 + 1G > A or del5395. Together they were not associated with the increased risk of breast cancer (North-Central control group: OR = 1.6, p = 0.124; the general Polish population: OR = 1.4, p = 0.109). This association was only seen for IVS2 + 1G > A mutation (OR = 3.0; p = 0.039). One of the three truncating *CHEK2* mutations (IVS2 + 1G > A, 1100delC, del5395) was present in 9 of 420 women diagnosed with breast cancer (2.1%) and in 4 of 121 women (3.3%) with a history of breast cancer in a first- and/or second- degree relatives. Together they were associated with the increased risk of disease in these groups, compared to the general Polish population (OR = 2.1, p = 0.053 and OR = 3.2; p = 0.044, respectively). I157T mutation was detected in 25 of 420 women diagnosed with breast cancer (6.0%) and in 8 of 121 women (6.6%) with a history of breast cancer in first- and/or second- degree relatives. The prevalance of I157T mutation was 4.1% (18/435) in North-Central control group and 4.8% (265/5.496) in the general Polish population. However it was not associated with an increased risk of breast cancer.

**Conclusion:**

Obtained results suggest that *CHEK2* mutations could potentially contribute to the susceptibility to breast cancer. The germline mutations of *CHEK2,* especially the truncating ones confer low-penetrance breast cancer predisposition that contribute significantly to familial clustering of breast cancer at the population level.

## Introduction

The genetic basis of breast cancer (BC) is very complex and it is suggested that many various genes, especially tumor suppressors could play role in disease development [1].

The *CHEK2* tumor suppressor gene belongs to the group of genes, which by regulating cell division protect the cells against too rapid, uncontrolled growth. In response to DNA damage Checkpoint kinase 2 (CHEK2) is activated on ATM-dependent pathway and then phosphorylates several substrates, such as p53, BRCA1 protein, CDC25A and CDC25C, involved in the cell cycle checkpoint control, through coordination of DNA repair, cell cycle progression and apoptosis [2].

Constitutional mutations of *CHEK2* predispose to many types of common cancers, e.g. breast cancer [3-5]. In the general Polish population the most common *CHEK2* mutations are I157T missense mutation and three premature protein-truncating mutations: IVS2 + 1G > A, del5395 and 1100delC, which prevalance is 4.8%, 0.4%, 0.4% and 0.2%, respectively [6].

In this report we present the results of research on association between congenital *CHEK2* mutations and a risk of BC in women originating from the North-Central Poland, as well as the relation of these mutations to familial history of BC.

## Materials and methods

### Patients

420 women from the North-Central Poland (Kujawy-Pomerania voivodeship) with consecutively diagnosed BC, treated in 2007-2010 at the Oncology Center in Bydgoszcz, were included in the investigation, regardless of histopatological type and family history of the BC. The median age at BC diagnosis in the whole group was 46 years (range 26-73). One woman was diagnosed with bilateral BC – two primary cancers within two years (age 41 and 42).

In families of women with congenital *CHEK2* mutations, molecular tests were performed. 61% (255/420) of women originated from families with at least one cancer in a close relative, the most frequently cancer of breast, ovary, lung, colon and prostate. 29% (121/420) of women originated from families with history of BC in first- and/or second- degree relatives. 5 out of 33 invited families (a total of 17 persons) agreed to be tested.

The first control group consisted of 435 healthy women from the North-Central region of Poland. The data on the general Polish population published by Cybulski et al. (2006) were used as the second control group (by courtesy of the Author) [6].

Informed consent was obtained from all patients and healthy persons. The study was approved by the Ethics Committee of the Collegium Medicum, Nicolaus Copernicus University in Bydgoszcz, Poland.

### Molecular analysis

The *CHEK2* mutations analysis was performed in all 420 patients and 17 members of their families, as well as in all women from the first control group.

The mutations were investigated in DNA from peripheral blood leukocytes, extracted by standard salting-out method. I157T and IVS2 + 1G > A were examined by RFLP-PCR, 1100delC by ASO-PCR, and del5395 by multiplex-PCR with two primer pairs flanking breakpoint sites in introns 8 and 10 [3,6].

Statistical analysis included the comparison of the frequency of variant alleles in studied and control groups. Odds ratios (ORs) were calculated from two-by-two tables, and statistical significance of differences between tested and control groups was estimated using the Fisher’s exact test.

## Results and discussion

At least one of *CHEK*2 mutations was found in 33 out of 420 women diagnosed with BC (7.9%). This frequency was higher than in both control groups, however differences were not statistically significant (first control group: 5.1%, p = 0.124; second control group: 5.8%, p = 0.109) (Table 1). The observed lack of statistically significant correlation between carrying of a congenital *CHEK2* mutation and the risk of BC in the patients from North-Central Poland may be caused by too small study groups.

**Table 1:** **The correlation between a constitutional *CHEK2 *** **mutation and a risk of BC in analyzed women**

***CHEK2 *** **mutations and groups of tested women**	**Carriers/total n = 420**		**Controls**^**a **^**n = 435**			**Controls**^**b **^**n = 5.496**		
			**OR**	**95% CI**	**p-Value**	**OR**	**95% CI**	**p-value**
**IVS2 + 1G > A**								
All women	5/420	(1.2%)	2.6	0.50-13.52	0.279	3.0	1.13-7.96	0.039^c^
Women from families with BC aggregation	1/121	(0.8%)	1.8	0.16-20.07	0.522	2.1	0.28-15.51	0.395
Controls^a^	2/435	(0.5%)	1.0					
Controls^b^	22/5.496	(0.4%)	1.0					
**1100delC**								
All women	2/420	(0.5%)	2.1	0.19-22.99	0.618	2.2	0.49-9.80	0.262
Women from families with BC aggregation	2/121	(1.7%)	7.3	0.66-81.14	0.121	7.7	1.70-34.70	0.035^c^
Controls^a^	1/435	(0.2%)	1.0					
Controls^b^	12/5.496	(0.2%)	1.0					
**del5395**								
All women	2/420	(0.5%)	2.1	0.19-22.99	0.618	1.1	0.26-4.63	0.707
Women from families with BC aggregation	1/121	(0.8%)	3.6	0.22-58.25	0.388	1.9	0.25-14.16	0.420
Controls^a^	1/435	(0.2%)	1.0					
Controls^b^	24/5.496	(0.4%)	1.0					
**Any protein truncating mutation**								
All women	9/420	(2.1%)	2.4	0.72-7.72	0.169	2.1	1.01-4.17	0.053^d^
Women from families with BC aggregation	4/121	(3.3%)	3.7	0.91-14.95	0.072^d^	3.2	1.14-8.98	0.044^c^
Women with no family history of BC	5/299	(1.7%)	1.8	0.49-6.88	0.498	1.6	0.63-4.01	0.140
Controls^a^	4/435	(0.9%)	1.0					
Controls^b^	58/5.496	(1.1%)	1.0					
**I157T**								
All women	25/420	(6.0%)	1.5	0.79-2.73	0.273	1.3	0.82-1.91	0.290
Women from families with BC aggregation	8/121	(6.6%)	1.6	0.70-3.87	0.327	1.4	0.68-2.90	0.286
Controls^a^	18/435	(4.1%)	1.0					
Controls^b^	264/5.496	(4.8%)	1.0					
**Total *CHEK2 *** **mutation**								
All women	33*/420	(7.9%)	1.6	0.91-2.80	0.124	1.4	0.94-1.99	0.109
Women from families with BC aggregation	12/121	(9.9%)	2.1	0.99-4.31	0.055^d^	1.8	0.96-3.24	0.076^d^
Controls^a^	22/435	(5.1%)	1.0					
Controls^b^	322/5.496	(5.8%)	1.0					

^*^Two mutations (I157T, IVS + 1G > A) in one woman with BC.

^a^Controls consisting of healthy women from North-Central region of Poland.

^b^Controls of the general Polish population, published by Cybulski et al. [6].

^c^Statistically important.

^d^Trend.

*OR* odds ratio.

*CI* confidence interval.

The I157T, 1100delC and del5395 mutations separately were disclosed with 6.0%, 0.5% and 0.5% frequency, respectively, but none of these frequencies was statistically significantly different from the frequency in both control groups (first control group: 4.1%, 0.2%, 0.2%, respectively; second control group: 4.8%, 0.2%, 0.4%, respectively). However, the prevalence of IVS2 + 1G > A mutation was three-fold higher in relation to the general Polish population and the presence of this mutation turned out to be associated with an increased risk of BC (1.2% vs. 0.4%; OR = 3.0; p = 0.039) (Table 1).

Reports of other authors show that the frequency of *CHEK2* mutations among women with diagnosed BC is different in various regions of Poland. Among BC women from throughout Poland and the South-Western Poland, these mutations were detected with higher frequency than in our investigations: 8.6% (385/4.454) and 9.9% (28/284), respectively [7,8].

The increased risk of BC associated with three *CHEK2* mutations IVS2 + 1G > A (1.1% vs. 0.48%; OR = 2.3, p = 0.04), 1100delC (0.5% vs. 0.25; OR = 2.0, p = 0.3) and I157T (6.7 vs. 4.8%; OR = 1.4, p = 0.02) was showed in 2004 by Cybulski et al. in BC women from the North-Western Poland and with four *CHEK2* mutations: IVS2 + 1G > A (1.0% vs. 0.4%; OR = 2.4, p = 0.0008), 1100delC (0.4% vs. 0.2%; OR = 2.1, p = 0.07), I157T (6.5% vs. 4.8%; OR = 1.4, p = 0.0004), del5395 (0.9% vs. 0.4%; OR = 2.0, p = 0.009) in 2007 by the same authors in a large group of BC women from throughout the country [3,7].

On the contrary, Myszka et al. (2011) did not show any relationship between the four investigated *CHEK2* mutations and the risk of BC among BC women from the South-Western Poland [8]. Such correlation was not found either by Kwiatkowska et al. (2006), who investigated only the 1100delC mutation in women with BC originating from East-Central, West-Central and South-Eastern Poland [9].

However, Han et al. (2013) in the meta-analysis of the risk of breast cancer associated with *CHEK2* I157T mutation in 15.985 patients from four countries (Bogdanova et al. 2005 [10]; Cybulski et al. 2004, 2009 [3,11]; Domagała et al. 2012 [12]; Dufault et al. 2004 [13]; Irmejs et al. 2006 [14]; Kleibl et al. 2008 [15]; Serrano – Fernandez et al. 2009 [16]) found a strong correlation between carrying of *CHEK2* I157T mutation and the risk of BC (OR = 1.58; p < 0.00001) [17].

In our study group any truncating *CHEK2* mutation occurred with two-fold higher frequency than in general Polish population and we have also found their association with the increased risk of BC (2.1% vs. 1.1%; OR = 2.1, p = 0.053) (Table 1). Cybulski et al. also observed a strong association of truncating mutations with elevated risk of BC in women from throughout the country (OR = 2.2; p = 0.0001) [7].

We also investigated the association between a positive familial history of BC in first- and/or second-degree relatives and the risk of developing BC. In women from these families we detected three-fold higher frequency of truncating *CHEK2* mutations together than in the general Polish population, and their association with the increased risk of BC (3.3% vs. 1.1%; OR = 3.2, p = 0.044). However, with regard to our control group, differences were at the limit of statistical significance (3.3% vs. 0.9%; OR = 3.7, p = 0.072), only. Among patients with truncating *CHEK2* mutations the risk of BC in the group of women from families with BC aggregation was two-fold higher than among women with no family history of BC (first control group: OR = 3.7 vs. OR = 1.8, second control group: OR = 3.2 vs. OR = 1.6) (Table 1).

In women from families with BC aggregation, 1100delC mutation frequency was eight and a half-fold higher in relation to the general Polish population and the presence of this individual mutation turned out to be associated with an increased risk of BC (1.7% vs. 0.2%; OR = 7.7; p = 0.035) (Table 1).

Cybulski et al. also showed a higher relative risk of BC among women with family history of BC, carriers of one of the three truncating mutations, from throughout the Poland compared to women with no family history of BC: 4.1%, OR = 5.0 (95% CI, 3.3 to 7.6) versus 2.8%, OR = 3.3 (95% CI, 2.3 to 4.7) [18].

The results of family investigations indicate a strong, statistically significant association of the truncating mutations with BC development, which suggests that mutations in *CHEK2* gene are associated with BC risk, especially in women originating from families with BC aggregation.

Our results are in favor of the results of Cybulski, and in contradiction with the results of Myszka and Kwiatkowska [3,7-9,18].

**Figure 1:**
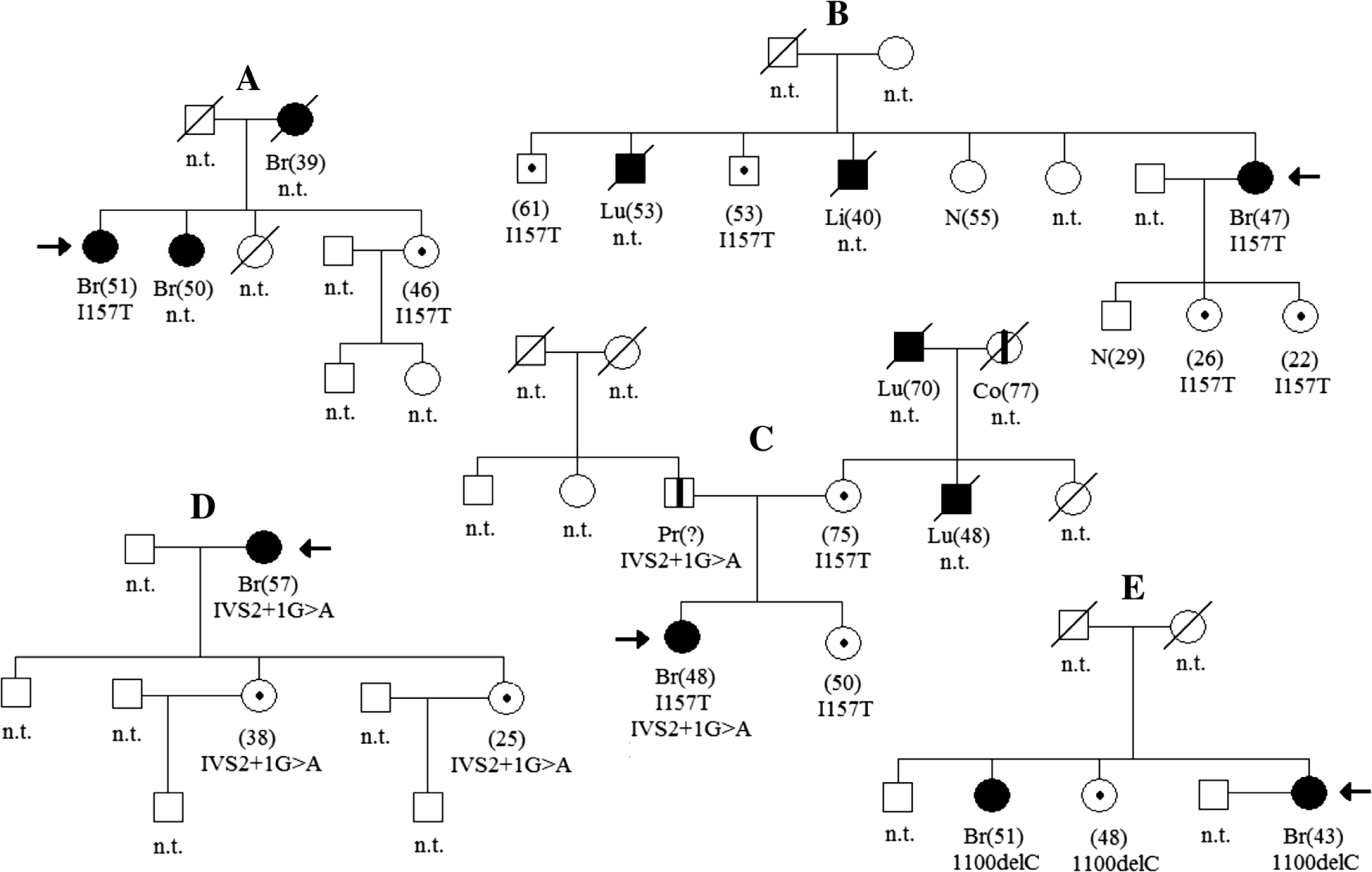
**Pedigrees of families A-E, with *CHEK2 *mutations.** Black symbols - persons affected with cancer; white symbols with a black dot inside - healthy mutation carriers. Age of cancer or age of *CHEK2* mutation diagnosis in healthy persons is given in brackets. Br - breast cancer; Lu - lung cancer; Li - liver cancer; Pr - prostate cancer; Co - colon cancer. Arrows indicate probands. I157T, IVS2 + 1G > A or 1100delC - mutation carriers; N - analyzed persons who are not carriers of the mutation; n.t. - not tested.

Our research, carried out in 5 families, confirmed hereditary character of all detected *CHEK2* mutations (Figure 1). In family A, I157T mutation was detected in two sisters: one with BC (age 51), and the second healthy (age at the time of examination 46). In family B, woman with BC at age 47 and her two healthy brothers (age 61 and 53), as well as her two healthy daughters (age 26 and 22), were carriers of I157T. Vertical transmission of I157T and IVS2 + 1G > A mutations was also observed in family C. I157T was detected in healthy mother (age 75) and two her daughters: one healthy (age 50), one with BC (age 48). BC-affected daughter inherited also second mutation, IVS2 + 1G > A, through paternal line. The father, carrier of IVS2 + 1G > A, was diagnosed with prostate cancer (age at diagnosis unknown). Family D was burdened with the IVS2 + 1G > A mutation, which was disclosed in mother (BC at age 57) and in her two healthy daughters (age 38 and 25). 1100delC was found in family E, in three sisters, two of which were diagnosed with BC (age 51 and 43).

In conclusion, we found a strong association between *CHEK2* truncating mutations prevalence and breast cancer family history. We conclude that *CHEK2* mutations are rare in BC, but our results suggest a tumor suppressor function of *CHEK2* gene in a small proportion of breast cancers. Furthermore, our results suggest that the *CHEK2* mutations are low-penetrance alleles with respect to BC. However, a variety of interactions between the mutated *CHEK2* with other genes can be related to the development of BC. The presence of *CHEK2* mutations in BC highlights the importance of the integrity of the DNA damage signals pathway in BC development. Although the mechanisms by which *CHEK2* mutations contribute to the development of BC remain unknown, future studies may confirm the observations shown in the present report.

## Abbreviations

ASO: Allele-specific oligonucleotide; ATM: Ataxia telangiectasia mutated; BC: Breast cancer; Br: Breast cancer; CDC25A: Cell division cycle 25 homolog A; CDC25C: Cell division cycle 25 homolog C; CHEK2: Cell-cycle checkpoint kinase 2; Co: Colon cancer; DNA: Deoxyribonucleic acid; Li: Liver cancer; Lu: Lung cancer; N: Analyzed persons who are not carriers of the mutation; n.t: Not tested; OR: Odds ratio; Pr: Prostate cancer; RFLP: Restriction fragment length polymorphism; p53: Tumor protein p53.

## Competing interests

The authors declare that they have no competing interests.

## Authors’ contributions

AB carried out the molecular genetic studies, performed the statistical analysis, wrote the manuscript. HJ conceived the study, participated in its design and coordination, and helped to draft the manuscript. AJ-C, MH and MP-D carried out the molecular genetic studies. RL and MP enrolled the patients into the study group. OH designed the study, participated in writing, critically revised the manuscript and approved its final version. All authors read and approved the final version of the manuscript.
